# Effects of repeated adolescent stress and serotonin transporter gene partial knockout in mice on behaviors and brain structures relevant to major depression

**DOI:** 10.3389/fnbeh.2013.00215

**Published:** 2013-12-31

**Authors:** Simona Spinelli, Tanja Müller, Miriam Friedel, Hannes Sigrist, Klaus-Peter Lesch, Mark Henkelman, Markus Rudin, Erich Seifritz, Christopher R. Pryce

**Affiliations:** ^1^Preclinical Laboratory for Translational Research into Affective Disorders, Department of Psychiatry, Psychotherapy and Psychosomatics, Psychiatric Hospital, University of ZurichZurich, Switzerland; ^2^Neuroscience Center, University and ETH ZurichZurich, Switzerland; ^3^Zurich Center for Integrative Human Physiology, University of ZurichZurich, Switzerland; ^4^Mouse Imaging Centre, Hospital for Sick ChildrenToronto, Canada; ^5^Division of Molecular Psychiatry, Department of Psychiatry, Psychosomatics and Psychotherapy, University of WürzburgWürzburg, Germany; ^6^Institute for Biomedical Engineering, University and ETH ZurichZurich, Switzerland; ^7^Department of Psychiatry, Psychotherapy and Psychosomatics, Psychiatric Hospital, University of ZurichZurich, Switzerland

**Keywords:** adolescent stress, serotonin transporter gene, magnetic resonance imaging, major depression, mouse model, development

## Abstract

In humans, exposure to stress during development is associated with structural and functional alterations of the prefrontal cortex (PFC), amygdala (AMY), and hippocampus (HC) and their circuits of connectivity, and with an increased risk for developing major depressive disorder particularly in carriers of the short (*s*) variant of the serotonin transporter (5-HTT) gene-linked polymorphic region (5-HTTLPR). Although changes in these regions are found in carriers of the *s* allele and/or in depressed patients, evidence for a specific genotype × developmental stress effect on brain structure and function is limited. Here, we investigated the effect of repeated stress exposure during adolescence in mice with partial knockout of the 5-HTT gene (HET) vs. wildtype (WT) on early-adulthood behavioral measures and brain structure [using magnetic resonance imaging (MRI)] relevant to human major depression. Behaviorally, adolescent stress (AS) increased anxiety and decreased activity and did so to a similar degree in HET and WT. In a probabilistic reversal learning task, HET-AS mice achieved fewer reversals than did HET-No-AS mice. 5-HTT genotype and AS were without effect on corticosterone stress response. In terms of structural brain differences, AS reduced the volume of two long-range white matter tracts, the optic tract (OT) and the cerebral peduncle (CP), in WT mice specifically. In a region-of-interest analysis, AS was associated with increased HC volume and HET genotype with a decreased frontal lobe volume. In conclusion, we found that 5-HTT and AS genotype exerted long-term effects on behavior and development of brain regions relevant to human depression.

## Introduction

Exposure to stressful life events during childhood and adolescence is considered to play a major role in increasing risks for developing neuropsychiatric disorders later in life (Mccrory et al., 2012). Neuroimaging studies in adults exposed to childhood maltreatment and neglect have consistently reported structural and functional abnormality in the prefrontal cortex (PFC), amygdala (AMY), and hippocampus (HC) (Hart and Rubia, 2012). These brain regions are known to be important in emotional regulation and stress reactivity and to be key structures in the pathophysiology of major depression (Price and Drevets, 2012), including reduced PFC and/or excessive AMY responses to the presentation of sad/fearful stimuli in depressed patients, in healthy individuals with high neuroticism and in healthy individuals exposed to childhood maltreatment (Cremers et al., 2010; Murray et al., 2011; Dannlowski et al., 2012). Differences in the responses to negative events and depression risk are also associated with predisposing genetic variants. In a seminal study, Caspi et al. (2003) reported that young adults exposed to repeated stressful life events were more likely to develop major depression if they were also carriers of the short (*s*) variant of the serotonin transporter gene-linked polymorphic region (5-HTTLPR) (Caspi et al., 2003). Although the findings have been questioned (Munafo et al., 2009; Risch et al., 2009), a recent meta-analysis confirms an increased risk of developing depression following childhood maltreatment in *s* allele carriers relative to *s* allele carriers not exposed to childhood maltreatment and to long (*l*) allele carriers (Karg et al., 2011). Importantly, the risk of major depression in carriers of the *s* allele of the 5-HTTLPR has been shown to increase as a function of the number of stressful events, suggesting that repeated stress may have a higher cumulative effect on brain development in these individuals.

In the absence of developmental stress, adult carriers of the *s* allele show reduced AMY and PFC volumes, and reduced fractional anisotropy (a measure of white matter integrity) in white matter tracts connecting the AMY and PFC as well as greater AMY response to fearful stimuli, relative to subjects homozygous for the *l* allele (Canli et al., 2005, 2006; Pezawas et al., 2005; Pacheco et al., 2009; Kobiella et al., 2011). However, evidence for a specific 5-HTTLPR × stress effect on brain structure and function is scarce. Frodl et al. (2010) found that emotional neglect during childhood was associated with smaller HC volume in adult depressed carriers of the *s* allele relative to *l/l* patients exposed to childhood neglect and to *s* carrier patients not exposed to neglect. The same study reports greater dorsolateral PFC volume in subjects homozygous for the *l* allele relative to *s* allele carriers independent of diagnosis (Frodl et al., 2010). A recent study reports that adolescents exposed to an adverse childhood environment and homozygous for the *s* allele were more sensitive to misleading negative feedback in a probabilistic reversal learning task than were *s/s* carriers not exposed to childhood adversity (Owens et al., 2012). The probabilistic reversal learning findings are noteworthy given that increased sensitivity to misleading negative feedback using the same task has also been reported in depression, and associated with abnormal PFC and AMY function (Murphy et al., 2003; Taylor Tavares et al., 2008). Overall, these results suggest an interaction between the 5-HTTLPR genotype and developmental stress exposure, which may modulate the development of the PFC leading to abnormal processing of negative feedback and an increased risk for major depression.

The effects of developmental stress exposure have been studied quite extensively in rodents. Most of these studies have been conducted in rats using different stress procedures in terms of stressor type, developmental stage, and duration of stressor exposure. In the majority of studies, the stress procedure was conducted during the first 3 weeks of life (Pryce and Feldon, 2003; Schmidt et al., 2011), a developmental phase that can be considered equivalent to a prenatal/early childhood period in humans (Clancy et al., 2007). However, stressful life events in humans are not limited to early childhood but rather occur across development (Nemeroff et al., 2003; De Bellis et al., 2010; Ressler et al., 2010). Consideration of the onset and the duration of the stress is likely to be particularly important because structural imaging studies in humans have clearly shown that different brain regions have different trajectories of development, suggesting region-specific windows of stress vulnerability (Tottenham and Sheridan, 2009; Giedd et al., 2010). Adolescence is considered a period of increased stress vulnerability for the PFC, which has a protracted development in both humans and rodents (Spear, 2000; Lupien et al., 2009), and thus a better understanding of the effects of adolescent stress (AS) exposure may be particularly relevant for major depression (Andersen and Teicher, 2008).

Recently, Schmidt et al. (2011) reviewed rodent studies investigating the effects of developmental stress procedures on depression-related behavioral and physiological measures, and concluded that a genetic background relevant to major depression in combination with stress exposure may be necessary for animal models to possess etiological validity (Schmidt et al., 2011). Causal evidence for rh-5-HTTLPR-*s* × early-life stress interaction has been obtained with the rhesus monkey: For example, in infant monkey carriers of the *s* allele, repeated social separation led to increased self-directed behaviors, considered a measure of “depression-like” behavior (Spinelli et al., 2012). In addition, higher cortisol in response to a stressor was reported in infant macaques carrying the *s* allele and raised by abusive mothers (Mccormack et al., 2009). These results indicate that the *s* allele increases stress sensitivity during early development such that several aversive episodes lead to depression-relevant phenotypes. A polymorphism orthologousto the human 5-HTTLPR is not present in rodents, but mice with a partial knockout for the 5-HTT gene (HET) show reduced levels of 5-HTT expression and function and, relative to wildtype (WT), represent a model of low 5-HTT function reported in human *s*-allele carriers relative to individuals homozygous for the *l*-allele (Murphy and Lesch, 2008). Adult HET mice that had not been previously stress-exposed exhibited increased fear conditioned freezing and increased learned helplessness following repeated exposure to an inescapable aversive stimulus (foot electro-shock) compared to WT mice (Pryce et al., 2012), in line with the increased reactivity to aversive stimuli exhibited by healthy human *s* carriers (Pezawas et al., 2005). Adult HET (non-stressed) mice actually exhibited decreased sensitivity to misleading negative feedback in comparison to WT mice in a rodent probabilistic reversal learning task (Ineichen et al., 2012). Studies of HET/WT × postnatal stress indicate that interactions between the HET genotype and stress are more often seen in terms of increased anxiety-like behaviors and in terms of molecular changes in the HC and the frontal cortex (Bartolomucci et al., 2010; Jansen et al., 2010; Heiming et al., 2011; Nietzer et al., 2011). Consistent with these findings in HET mice, adult 5-HTT full knockout (KO) mice (non-stressed) show increased anxiety-related behaviors (Carroll et al., 2007), higher depression-related behaviors when exposed to repeated stress and decreased fear extinction [(Wellman et al., 2007) see also (Carola and Gross, 2012; Homberg and Van Den Hove, 2012)]. In adult rats, 5-HTT KO leads to increased anxiety- and depression-related behaviors, whereas absence of 5-HTT is protective against construction stress (Schipper et al., 2011). Notably, these studies have investigated the effects of stress during either the first 3 weeks of life or adulthood. In terms of brain studies, a previous comparison of KO vs. WT mice reported differences in histology that were not identified using structural magnetic resonance imaging (MRI) (Bearer et al., 2009). However, structural MRI has not been used to specifically investigate the effects of developmental stress in HET vs. WT mice.

The aim of the present study was to assess the effects of repeated stress exposure during adolescence in HET and WT mice in terms of: (1) anxiety- and depression-relevant behaviors and (2) development of the frontal lobe, AMY, and HC. We hypothesized that: (1) AS and HET genotype would lead singly and additively to long-term increased anxiety in behavioral tests thereof; (2) AS would lead to reduced performance in a probabilistic reversal learning task in both WT and HET mice; (3) AS and HET genotype would lead singly and additively to reduced volumes of the frontal lobe, AMY, and HC.

## Materials and methods

### Animals

Male and female mice of a 5-HTT null mutant strain on a C57BL/6J background (>20 backcross generations) were transferred from the University of Würzburg (Bengel et al., 1998) and breeding was established in-house with WT dams and HET sires. Mice were under a reversed 12:12 h light-dark cycle with temperature at 20–22°C and humidity at 50–60%. Ninety-eight male mice were used in the study. At the age of 4 weeks, mice were weaned and housed as brother-pairs in individually-ventilated cages (type 2 long) containing sawdust, a sleeping house and bedding, with *ad libitum* food and water. Behavioral training and testing was carried out under dimmed light conditions during the dark phase. All procedures were conducted under a permit for animal experimentation issued by the Veterinary Office, Zurich, Switzerland in accordance with the Animal Protection Act (1978) Switzerland. All efforts were made to minimize the number of mice used and any extraneous stress of those mice that were used.

### Experimental design

Each brother-pair in the same cage was randomly allocated either to the AS group or to the control (No-AS) group and studied in one of two experiments. The AS/No-AS manipulation was performed between postnatal days (PND) 28 and 47, and mice were studied in readout tests across late adolescence and early adulthood.

Experiment 1 included 44 mice (HET-No- AS = 12; HET-AS = 10; WT-No-AS = 10; WT-AS = 12) that were tested at PND 54–59 in the novelty induced hypophagia test and 1 day later in the elevated plus maze test, to assess anxiety. At PND 80–84, a serial blood sampling procedure was conducted to assess reactivity of the hypothalamic-pituitary-adrenal-axis. Between PND 92 and 150, mice were trained and tested in a probabilistic reversal learning task.

Experiment 2 included 54 mice that were tested at PND 64–69 in the open field to assess activity and anxiety. At PND 80–83, the brains were fixed and studied using structural MRI. Three subjects were excluded due to a problem with the realignment procedure, so that the final sample size was HET-No-AS = 14; HET-AS = 12; WT-No-AS = 12; WT-AS = 13.

**Figure 1:**
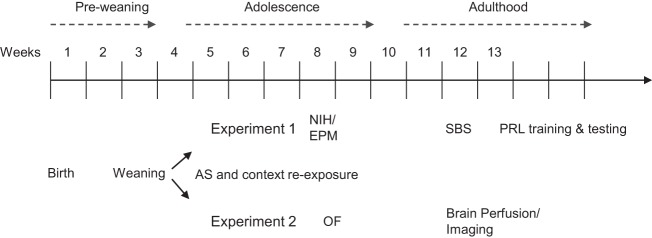
**The time line of the study**. Animals were weaned at postnatal week 4 and assigned to experiment 1 or 2. All mice were exposed to the adolescence stress procedure and context re-exposure. Mice in experiment 1 were subsequently tested during late adolescence in the novelty-induced hypophagia (NIH) and elevated plus maze (EPM) tests. In adulthood, a serial blood sampling (SBS) procedure was conducted, afterwards animals were trained and tested in the probabilistic reversal learning (PRL) task. Mice in experiment 2 were tested during late adolescence in the open field (OF), and perfused in adulthood to collect the brains for structural magnetic resonance imaging.

The time lines of experiments 1 and 2 are detailed in Figure 1.

### Adolescent stressor

The stress procedure consisted of nine sessions of electro-shock exposure (S1–S9). S1 and S9 were always conducted at PND 28 and 47, respectively; otherwise, three sessions were conducted per week and sessions S2–S8 were distributed regularly between PND 29 and 46, with one session per day and two consecutive daily sessions maximum. Each session consisted of 24 electro-shocks of variable duration (2–5, mean: 3 s), administered on a variable interval schedule (12–108, mean: 60 s). The electro-shock intensity (mA) increased across S1–S9 to reduce habituation to the stress procedure: 0.1 (S1), 0.15 (S2), 0.2 (S3), 0.25 (S4 and S5), and 0.3 (S6–S9). Mice in the No-AS group were placed in the environment with an electro-shock intensity of 0. The total locomotor distance (arbitrary units) and total freezing duration during the intervals between electro-shocks were measured.

Stressor sessions were conducted using a fully-automated apparatus (Multi Conditioning System, TSE Systems GmbH, Bad-Homburg, Germany) as described in (Pryce et al., 2012). Behavioral measures were motor activity in arbitrary units and duration of freezing episodes defined as no detection of any movement for a minimum of 2 s.

### *In vivo* behavioral and physiological tests

#### Adolescent stress context re-exposure

On PND 49, 2 days after S9, mice were re-exposed to the AS/No-AS arena for a period of 20 min without electro-shocks. Percent time spent freezing was analyzed in 4-min intervals.

#### Novelty-induced hypophagia (NIH) test

The NIH test was adapted from (Bechtholt et al., 2007). Animals were trained in the holding room and home cage to consume a palatable food (Frosted Flakes, Migros, Zürich, Switzerland) on 2 consecutive days. Thereafter, mice were habituated to the home cage testing procedure: to avoid the stress of single-caging, a Plexiglas divider was introduced in the home cage 30 min before the beginning of habituation and testing sessions. Mice were not food deprived before testing. One flake positioned on a plastic dish of 2 × 2 cm was presented to each animal separately for a maximum of 20 min. Six animals never ate the flake and were excluded from further analysis. On the evening of day 5, mice were moved to a dedicated testing room. On day 6, the latency to eat the familiar palatable food in the home cage was measured. The NIH test was conducted on day 7 under dim light (2 lux), with subjects aged PND 54–59: The test cage had the same dimensions as the home cage; it was without shavings. The latency to eat the familiar palatable food in the novel environment was measured; if a subject did not eat the food within 30 min the session was stopped and a latency of 1800 s assigned.

#### Elevated plus maze (EPM) test

The EPM test was conducted 1 day after the NIH test in a dedicated room under low light intensity (5 lux at the end of the open arms). The apparatus comprised two closed arms enclosed by walls 15(H) cm, two open arms (OA) 30(L) × 5(W) cm and a center (5 × 5 cm) quadrant, elevated on legs [50(H) cm]. The EPM test was conducted. The mouse was placed on the center of the maze facing an open arm. Entries into and time spent on the open and closed arms were measured during 5 min using a video-tracking system (VideoMot 2, TSE Systems GmbH). Total arm entries and distance moved were used as indices of locomotion; the ratio of OA entries to total arm entries and the ratio of time spent in the OA to total time spent on arms, were used as indices of anxiety.

#### Open field (OF) test

The test was conducted between PND 64 and 69 in mice that were subsequently studied in the structural brain analysis (Experiment 2). The OF measured 50 × 50 × 30(H) cm; the test was conducted under high illumination (50 lux in the center of the arena). After overnight habituation to the test room, mice were placed into the center of the OF. Mouse location and movement were monitored using a video-tracking system (VideoMot 2, TSE Systems GmbH). The OF was divided virtually into center (30 × 30 cm) and periphery. The OF test duration was 30 min and data were analyzed in 5-min bins. Data for one mouse (WT-No-AS) could not be used due to a technical problem. Total distance moved was used as an index of activity and percent time spent in the center was used as a measure of anxiety i.e., greater avoidance of center equals greater anxiety.

#### Serial blood sampling (SBS)

At PND 80–84, SBS was performed as described in (Pryce et al., 2011b). To ensure that corticosterone titers were at baseline at sampling onset, per brother-pair one mouse was sampled per day on two consecutive days. During the second-half of the dark/active period (12:00–19:00 h), the mouse was placed in a plastic restrainer (10 L × 4Ø cm, Indulab, Gams, CH) and an incision was made in the lateral tail vein to collect 60 μl of blood into an EDTA-coated capillary blood tube (Microvette 500, Sarstedt). Samples were collected at 0 (baseline), 20, 60, and 120 min intervals from the same incision site. Between successive blood samplings the mouse was returned to the home cage. All samples were placed on ice and centrifuged at 4°C followed by removal and storage of plasma at −80°C until hormone determination. Corticosterone levels were measured using ELISA kit (AssayMax, AssayPro, St. Charles, USA). Insufficient volume was collected from one WT-AS mouse.

#### Probabilistic reversal learning (PRL) task

The mouse PRL test apparatus and training and testing procedures is described in (Ineichen et al., 2012) and Supplementary Figure 1. Mice were trained and tested in adulthood, aged between PND 92–96 at onset of training and between PND 130–150 at testing. In brief, 1 week before the beginning of PRL training, mice were food restricted to 90% of their free-feeding body weights and maintained at this weight throughout training/testing. Training and testing were carried out in operant boxes (TSE Systems GmbH). The nose-poke ports were situated to the left and right of a feeder element, into which sucrose pellets (14 mg, Dustless Precision Pellets, TSE System GmbH) were delivered singly. A correct response initiated a tone (conditioned stimulus, CS) from a speaker located above the feeder port and pellet delivery, and pellet retrieval was detected via infra-red beam. Three animals could not be trained to criterion on the final PR stages (one each from HET-AS, WT-AS, WT-No-AS groups). Mice were trained to a high level of proficiency in serial reversal before proceeding to the PRL task [see Ineichen et al. (2012) for learning criteria for reversal training].

The test conditions were a maximum of 60 rewards or 30 min and on each trial the mouse could make an operant response to the left or right nose-poke port, with one nose-poke set to correct (sucrose pellet) and one set to incorrect/punish (no pellet + 5 s time out). After eight consecutive correct trials the nose-port—outcome contingency was reversed. In addition to the reversal schedule (a maximum of seven reversals were possible), a proportion of correct responses were punished. The probability of punished correct responses was set to 0.1 or 0.2. Additional test settings were that the first correct response per session was never punished and the maximum number of consecutive punished correct responses was set to 2. The evidence suggests that at 0.1 punished correct responses mice maintain accurate reward expectancy and punishment expectancy (Ineichen et al., 2012). Measures of interest were: session duration; total number of reversals completed; probability of reward-stay responding (p Reward-Stay), defined as trials with mouse responding to port that was correct on previous trial/total trials immediately following a correct trial; probability of negative feedback sensitivity (p NFS), defined as trials with mouse responding to the opposite port to that at which a correct response was punished on previous trial/total trials immediately following a punished correct response; post-reinforcement latency, defined as the latency to make an operant response after collection of the previous reward; % trials with long post-reinforcement latency, with long post-reinforcement latency defined as greater than the mean +1.5 *SD* (calculated from the post-reinforcement latencies of all mice).

### *In vivo* data analysis

Statistical analyses were conducted using StatView 5.0.1 (SAS Institute, Inc., Cary, NC). All data are reported as mean ± standard error of the mean (s.e.m.), with significance set at *p* < 0.05 two-tailed alternatives. The body weight and EPM data were analyzed using analysis of variance (ANOVA) with stress exposure (AS, No-AS) and 5-HTT genotype (WT, HET) as between subject factors. The AS procedure, OF and PRL data, as well as plasma corticosterone titers and body weight, were analyzed using repeated measures ANOVA (RM-ANOVA) with stress exposure (AS, No-AS), and genotype (WT, HET) as between subject factors and session or interval as within subject factors. For the NIH data, a survival analysis according to the Kaplan-Meier and log-rank (Mantel-Cox) tests was conducted; Cox proportional hazards regression model was used to compare the relative influences of stress exposure (AS, No-AS) and genotype (WT, HET) on latency to eat.

### Brain perfusion

At PND 80–83, mice were anesthetized with pentobarbital-Na injected i.p. and transcardially perfused at a rate of 1 ml/min with 30 ml phosphate-buffered saline (PBS, pH7.4) containing 2 mM ProHance®(gadoteridol, Bracco Diagnostics Inc., Princeton, NJ) contrast agent solution at room temperature (25°C), followed by 30 ml ice-cold 4% paraformaldehyde in PBS/2 mM ProHance®. The heads were removed and the skin, lower jaw, ears and cartilaginous nose tip were removed from the skull. The brains in the remaining skull structures were fixed in 4% paraformaldehyde containing 2 mM ProHanceat 4°C for 24 h. The skulls were then transferred to PBS with 0.02% sodium azide and 2 mM ProHance® solution at 4°C until they were scanned (Cahill et al., 2012).

### *In vitro* MRI

A multi-channel, 7 T, 40 cm diameter bore magnet (Varian Inc. Palo Alto, CA) was used to acquire anatomical images. A custom-built 16-coil solenoid array was used to image 16 samples concurrently (Dazai et al., 2011). Parameters used in the scan were optimized for gray–white matter contrast: a T2-weighted 3D fast spin-echo sequence, with TR = 2000 ms, echo train length = 6, TE eff = 42 ms, field-of-view (FOV) = 25 × 28 × 14 mm and matrix size = 450 × 504 × 250, giving an image with 56 μm isotropic voxels. In the first phase-encode dimension, consecutive k-space lines were assigned to alternating echoes to move discontinuity-related ghosting artifacts to the edges of the FOV (Thomas et al., 2004). This scheme necessitates oversampling in the phase-encode direction to avoid interference of the ghosts with the main image. This first phase-encode was oversampled by a factor of 2 (504 phase-encode points) giving a FOV of 28 mm that was subsequently cropped to 14 mm after reconstruction. Total imaging time was 11.7 h. T2 relaxation maps were acquired for both a medial axial and sagittal slice using a multi-slice spin-echo sequence with 11 different TEs ranging from 0.008 to 0.1 s. The imaging parameters used were TR = 2000 ms, FOV = 28 × 14 mm, matrix size = 256 × 128, slice thickness = 0.5 mm and 2 averages.

### Image processing and analysis

All registrations were performed using a combination of the mni_autoreg tools (Collins et al., 1994) and ANTS (Avants et al., 2010). The processing and analysis procedures are detailed elsewhere (Kovacevic et al., 2005; Lerch et al., 2011). All scans were first registered linearly and then non-linearly together, and subsequently re-sampled to create a population atlas representing the average anatomy of the study sample. The end result was to have all scans deformed into exact alignment with each other in an unbiased fashion. As part of this process, a deformation field was calculated for each mouse. This deformation field was a transformation from the individual mouse's anatomy to the final atlas space (Nieman et al., 2006; Lerch et al., 2008). The Jacobian determinants of the deformation fields were then calculated as measurements of volume differences at each voxel. To calculate volume changes in specific brain regions, a pre-classified atlas dividing the brain into 62 separate structures was aligned onto the study population-specific atlas (Dorr et al., 2008). The resulting atlas was then used in conjunction with the Jacobian determinants to calculate volumes for each region in the brain and for all individual brains in the study. In addition, more-localized changes were examined by comparing Jacobian determinants on a per voxel basis. Because of the millions of separate statistical tests required for this type of analysis, inflated type I error was corrected for using a 10% False Discovery Rate [FDR, (Genovese et al., 2002)] for both the anatomical and voxel-wise analyses. In addition to the whole brain analysis, region-of-interest analyses of the frontal lobe, AMY, and HC were conducted.

## Results

### *In vivo* behavior and physiology

#### Physical development

In Experiment 1, mice showed no significant difference in body weight between groups at any age. In Experiment 2, there was a significant genotype × AS interaction effect prior to AS group allocation [*F*_(1, 47)_ = 22.5, *p* < 0.007]; a posteriori pair-wise comparisons demonstrated that body weight (g) was higher in WT-No-AS compared to HET-No-AS (*t* = 2.8, *p* < 0.02) and to HET-AS mice [*t* = 3.4, *p* < 0.003 (HET-No-AS: 15.1 ± 0.3, HET-AS:15.5 ± 0.6, WT-No-AS: 16.6 ± 0.4, WT-AS: 14.3 ± 0.5). This interaction was still present during context re-exposure (*F*_(1, 47)_ = 4.5, *p* < 0.04): HET-No-AS: 23.5 ± 0.2, HET-AS: 23.4 ± 0.5, WT-No-AS: 24.3 ± 0.3, WT-AS: 22.7 ± 0.4]. However, there was no significant difference when the mice were tested in the OF (PND 64–69) and thereafter.

#### Adolescent stress procedure

**Figure 2:**
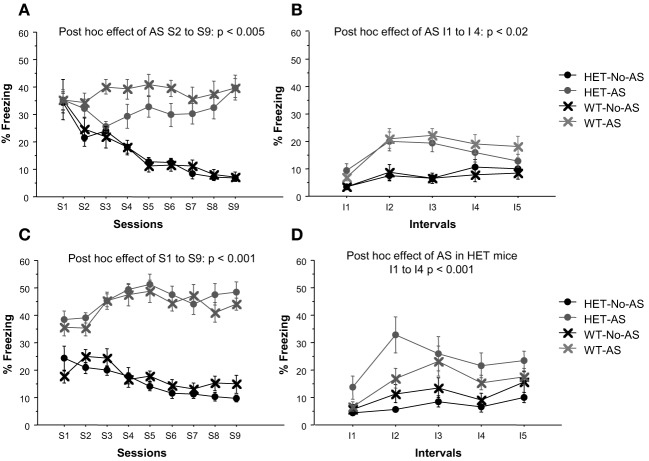
**Results of the adolescent stress procedure on freezing behavior**. Mean percent time spent freezing during the adolescent stress sessions (S1–S9) for mice in experiment 1 **(A)** and 2 **(C)**. Mean percent time spent freezing during the context re-exposure over 5 intervals (I) of 4-min each for mice in experiment 1 **(B)** and 2 **(D)**. Results are reported as mean ± s.e.m.

In Experiment 1, there was a significant AS × session interaction effect on % time freezing [*F*_(8, 320)_ = 13.4, *p* < 0.0001, Figure 2A]. *A posteriori* analysis of each stress session separately showed that there was no effect of AS at S1 (*p* > 0.9) and thereafter there was a monotonic decrease in % time freezing in No-AS mice specifically resulting in increased % time freezing in AS vs. No-AS mice (S2: *p* < 0.005; S3–S9: *p* < 0.001). There was no significant effect involving genotype. Also in Experiment 2, there was a significant AS × session interaction on % time freezing [*F*_(8, 376)_ = 10.9, *p* < 0.0001, Figure 2C] and no effect of genotype. *A posteriori* analysis of each session separately demonstrated a significant increase in % time freezing in AS vs. No-AS mice at all sessions (S1–S9: *p* < 0.001).

#### Adolescent stress context re-exposure

In Experiment 1, there was a significant AS × interval interaction for % time freezing [*F*_(4, 160)_ = 3.8, *p* < 0.006, Figure 2B]. *A posteriori* analysis showed that % time freezing was greater in AS than in No-AS mice at each interval (I1–I5: *p* < 0.04). No significant effect of genotype was found. In Experiment 2, there was a significant genotype × AS interaction effect on % time freezing [*F*_(1, 47)_ = 4.94, *p* < 0.04, Figure 2D]: in HET mice, mean-session % time freezing was significantly higher in AS compared to No-AS mice [*t*_(24)_ = 4.2, *p* < 0.001] and in WT mice there was no AS effect.

#### Novelty-induced hypophagia test

**Figure 3:**
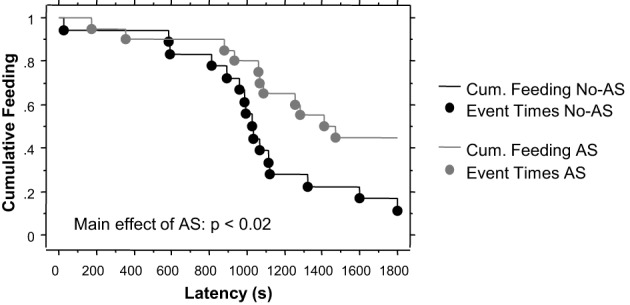
**Results of the novelty-induced hypophagia test (experiment 1)**. Lines indicate the cumulative feeding over a 30 min session (1800 s) and dots the time point at which the feeding (event) occurred. No effect of genotype was found.

The survival analysis of latency to eat palatable food showed a significant effect of AS (Chi-Square = 5.5, *p* < 0.02). As shown in Figure 3, 45% of AS mice failed to eat the palatable food compared to 11% of No-AS mice. There was no effect of genotype, including when AS and No-AS groups were analyzed separately (*p* > 0.4). No significant differences were found when mice were tested in the home cage: all mice ate the palatable food within 193 s excluding one outlier in the HET-AS group that needed 698 s (Latency: HET-No-AS: 66.8 ± 17.4, HET-AS: 107.3 ± 65.8, WT-No-AS: 59.9 ± 12.3, WT-AS: 79.3 ± 13.4).

#### Elevated plus maze test

**Figure 4:**
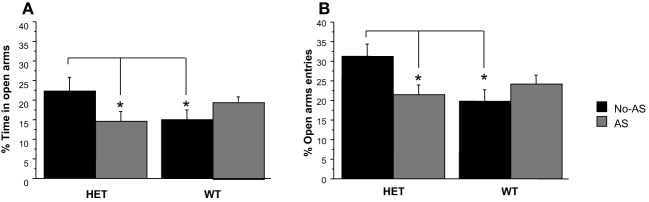
**Results of the elevated plus maze test (experiment 1)**. Mean percent time spent in the open arm **(A)** and mean percent open arm entries **(B)**. Percent open arm entries was lower in HET-AS mice relative to HET-No-AS mice and WT-AS mice (^*^p < 0.03). Results are reported as mean ± s.e.m.

For % time on OA, there was a significant genotype × AS interaction [*F*_(1, 40)_ = 5.6, *p* < 0.03, Figure 4A]; *a posteriori t*-tests revealed that the largest pair-wise effect was a non-significant tendency for HET-No-AS mice to spend greater % time on OA than HET-AS mice (*t* = 1.7, *p* < 0.09). For % OA entries, there was a significant genotype × AS interaction [*F*_(1, 40)_ = 6.6, *p* < 0.02, Figure 4B]: *a posteriori t*-tests demonstrated that HET-AS mice exhibited reduced % OA entries relative to HET-No-AS mice [*t*_(20)_ = 2.4, *p* < 0.03] and WT-AS mice [*t*_(20)_ = 2.6, *p* < 0.02]. For total number of entries there was a significant genotype effect [*F*_(1, 40)_ = 4.8, *p* < 0.04]; WT mice made more total arm entries than HET mice (HET-No-AS: 24.5 ± 1.0, HET-AS: 20.8 ± 0.5, WT-No-AS: 24.8 ± 0.4, WT-AS: 24.2 ± 0.3). For total distance moved there was a significant main effect of genotype [*F*_(1, 40)_ = 5.4, *p* < 0.03], with WT mice traveling a greater distance than HET mice (data not shown).

#### Open field test

**Figure 5:**
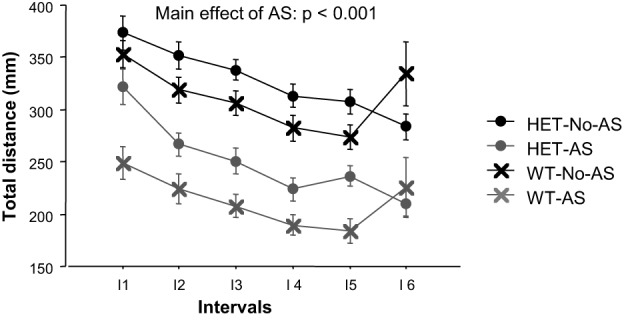
**Total distance moved during the open field test (experiment 2) over six intervals (I) of 5 min each**. Results are reported as mean ± s.e.m.

For total distance moved in the OF, there was a main effect of AS [*F*_(1, 46)_ = 19.9, *p* < 0.001, Figure 5] with AS mice exhibiting reduced activity relative to No-AS mice. The main effect of interval [*F*_(5, 230)_ = 9.2, *p* < 0.0001] was due to the monotonic decrease in activity by all groups across test intervals. The % distance moved in the center increased over time as indicated by a main effect of interval [*F*_(5, 220)_ = 33.3, *p* < 0.0001; data not shown]. There was no significant effect involving AS or genotype on % distance moved in the center.

#### Serial blood sampling

**Figure 6:**
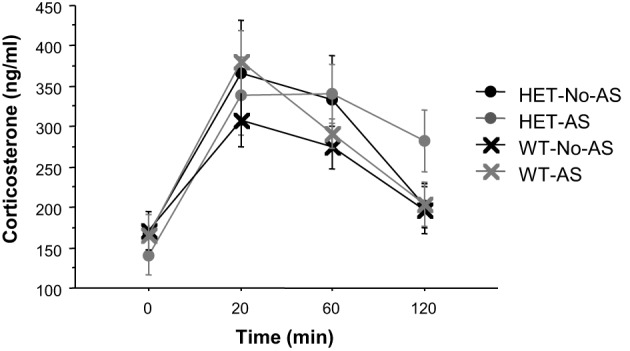
**Corticosterone titers during the serial blood sampling procedure (experiment 1)**. Results are reported as mean ± s.e.m.

For plasma corticosterone (CORT) titers there was a significant main effect of sample [*F*_(3, 117)_ = 34.9, *p* < 0.001] which was due to a lower plasma CORT titers at 0 min relative to 20, 60, and 120 min (Figure 6). There was no effect involving AS or genotype (*p* > 0.2). There was also no significant effect of AS or genotype on delta CORT values for time at 20 minus 120 min (*p* > 0.3), indicating a similar recovery of CORT concentrations to basal levels across groups.

#### Probabilistic reversal learning task

Table 1 gives the PRL data for the session that used probability 0.1 for punished correct responses. All mice obtained the maximum number of rewards (60). For number of reversals completed there was a significant genotype × AS interaction [*F*_(1, 37)_ = 4.5, *p* < 0.05]: *a posteriori* pair-wise comparisons demonstrated that HET-AS mice completed fewer reversals than HET-No-AS mice [*t*_(19)_= 2.4; *p* < 0.03]. For session duration there was a significant genotype × AS interaction [*F*_(1, 37)_ = 4.3, *p* < 0.05]: *a posteriori* pair-wise comparisons demonstrated that session duration was longer in HET-AS mice than HET-No-AS mice [*t*_(19)_= 2.4; *p* < 0.03] and no significant pair-wise differences involving WT mice. This potential indication of reduced task motivation was investigated using additional measures: for mean post-reinforcement latency (s) there was a borderline non-significant AS × genotype interaction [*F*_(1, 37)_ = 3.3, *p* < 0.08]: HET-AS mice (6.1 ± 0.8) had a longer post-reinforcement latency than HET-No-AS mice [4.0 ± 0.4; *t*_(19)_ = 2.4, *p* < 0.03]. In terms of % trials with a long post-reinforcement latency (>13 s, corresponding to the mean +1.5 *SD* as calculated from the post-reinforcement latencies of all mice), there was a significant genotype × AS interaction [*F*_(1, 37)_ = 4.5, *p* < 0.05]: HET-AS mice had a higher score for this measure (4.4 ± 1.4 %) compared to HET-No-AS mice [1.2 ± 0.6 %; *t*_(19)_ = 3.0, *p* < 0.009]. As given in Table 1, there was no effect involving AS or genotype on the two other primary measures of the PRL task, namely p reward-stay and p NFS. Also, there were no effects involving AS or genotype when a probability of 0.2 was used for punished correct responding (data not shown).

**Table 1:** **Performance on the probabilistic reversal task with 10 % probability of punished correct responses**.

**PRL measures**	**HET-No-AS**	**HET-AS**	**WT-No-AS**	**WT-AS**	**AS**	**Genotype**	**Genotype × AS**
Number of trials completed	60	60	60	60	n.s.	n.s.	n.s.
Number of reversals	5.33 ± 0.23^a^	4.33 ± 0.37^b^	4.89 ± 0.31	5.27 ± 0.38	n.s.	n.s.	*p* < 0.05; a vs. b
p reward-stay	0.85 ± 0.02	0.82 ± 0.02	0.83 ± 0.02	0.85 ± 0.03	n.s.	n.s.	n.s.
p NFS	0.69 ± 0.06	0.58 ± 0.05	0.60 ± 0.06	0.56 ± 0.06	n.s.	n.s.	n.s.
Session duration (s)	636.00 ± 44.00^a^	896.67 ± 111.03^b^	770.67 ± 81.05	705.82 ± 78.37	n.s.	n.s.	*p* < 0.05; a vs. b
Post-reinforcement latency (s)	4.0 ± 0.4^a^	6.1 ± 0.9^b^	4.8 ± 0.8	4.5 ± 0.7	n.s.	n.s.	*p* < 0.08; a vs. b
% trials with long post-reinforcement latency	1.2 ± 0.6^a^	4.4 ± 1.4^b^	3.0 ± 1.2	2.4 ± 0.7	n.s.	n.s.	*p* < 0.05; a vs. b
Number of errors per reversals	1.66 ± 0.21	1.32 ± 0.15	2.09 ± 0.30	1.89 ± 0.32	n.s.	*p* < 0.07	n.s.
Number of errors after reversal	10.83 ±1.79	13.67 ± 1.56	13.00 ± 1.55	11.46 ± 2.41	n.s.	n.s.	n.s.
Feeder response latency (ms)	626.58 ± 19.95	1421.28 ± 652.65	724.00 ± 75.95	1071.96 ± 428.92	n.s.	n.s.	n.s.
Number of feeder responses	99.75 ± 3.85	108.33 ± 6.18	98.44 ± 5.38	108.18 ± 7.14	n.s.	n.s.	n.s.

Results are reported as mean ± s.e.m, n.s.: not significant.

### *In vitro* MRI

#### Whole brain analysis

**Figure 7:**
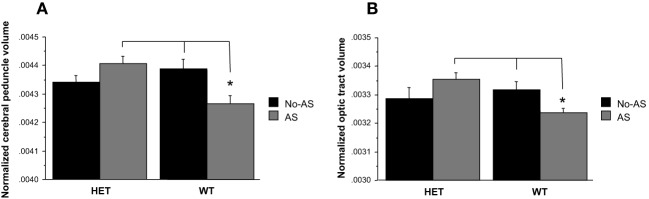
**Analysis of the normalized volumes of 62 structures showed that a significant effect of AS in two white matter regions: the cerebral peduncle (10% FDR) and the optic tract (5% FDR)**. WT-AS mice showed a smaller cerebral peduncle **(A)** and optic tract **(B)** volumes relative to WT-No-AS mice and to HET-AS mice (^*^p < 0.02). Results are reported as mean ± s.e.m.

Total brain volume showed no significant differences between groups (Table 2, AS: *p* > 0.9, genotype *p* > 0.7, genotype × AS: *p* > 0.9). Analysis of the normalized volumes of all 62 structures showed that AS led to a significant volume decrease in two white matter regions, the optic tract (OT) at 5% FDR and the cerebral peduncle (CP) at 10% FDR. To assess whether this effect was associated with a genotype × AS interaction, separate RM-ANOVAs were conducted on the normalized volumes of the OT and CP. A significant interaction was found for the OT [*F*_(1, 47)_ = 6.9, *p* < 0.02, Figure 7A] and the CP [*F*_(1, 47)_ = 10.5, *p* < 0.003, Figure 7B]. For OT and CP, WT-AS mice exhibited reduced CP volume relative to WT-No-AS mice [*t*_(23)_ = 2.6, *p* < 0.02; *t*_(23)_ = −2.7, *p* < 0.02; respectively] and to HET-AS mice [*t*_(23)_ = 4.3, *p* < 0.0003; *t*_(23)_ = 3.5, *p* < 0.003; respectively]. The interactions did not survive correction for multiple comparisons in the whole brain analysis.

**Table 2:** **Absolute volumes of anatomical measures**.

**Volumes (mm3)**	**HET-No-AS**	**HET-AS**	**WT-No-AS**	**WT-AS**	**AS**	**Genotype**	**Genotype × AS**	**Hemisphere**
Total brain	473.79 ± 2.53	474.09 ± 3.15	475.07 ± 3.87	475.11 ± 3.37	n.s.	n.s.	n.s.	-
Frontal lobe	49.08 ± 0.36	48.52 ± 0.62	49.72 ± 0.47	50.57 ± 0.59	n.s.	*p* < 0.008	n.s.	-
Right amygdala	7.48 ± 0.06	7.56 ± 0.10	7.63 ± 0.06	7.55 ± 0.11	n.s.	n.s.	n.s.	*p* < 0.001
Left amygdala	7.15 ± 0.05	7.11 ± 0.09	7.25 ± 0.05	7.12 ± 0.08	n.s	n.s.	n.s.	-
Right hippocampus	9.97 ±0.12	10.20 ± 0.09	10.04 ± 0.13	10.15 ± 0.10	*p* < 0.04	n.s.	n.s.	n.s
Left hippocampus	10.03 ± 0.09	10.13 ± 0.09	10.18 ± 0.10	10.15 ± 0.09	n.s.	n.s.	n.s.	-

Results are reported as mean ± s.e.m. and p values are calculated for normalized volumes (region volume/total brain volume), n.s.: not significant.

**Figure 8:**
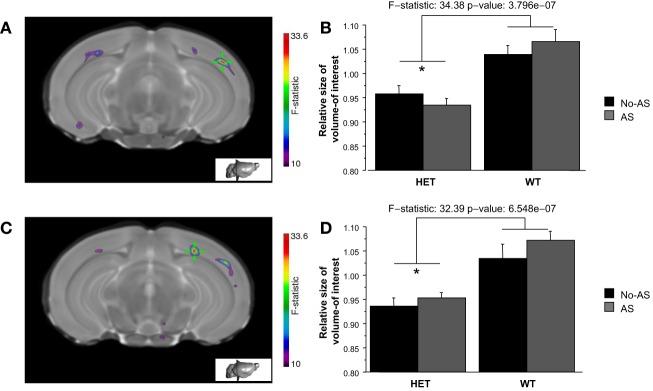
**The whole brain voxel-wise analysis showed a significant effect of genotype (10% FDR) in two clusters of right temporal-parietal cortex (A and C)**. HET mice showed tissue volume reduction relative to WT mice (**B** and **D**, ^*^p < 0.00001). Results are reported as mean ± s.e.m.

Volumetric differences across the brain were also examined on a voxel-wise basis in order to increase statistical sensitivity for detection of more localized changes. The voxel-wise analysis of the statistical map of the Jacobian determinant indicated a significant effect of genotype at 10% FDR in two clusters of the right temporal-parietal cortex (Figures 8A,C) with HET mice showing a tissue volume reduction compared to WT mice (Figures 8B,D).

#### Region-of-interest analysis

**Figure 9:**
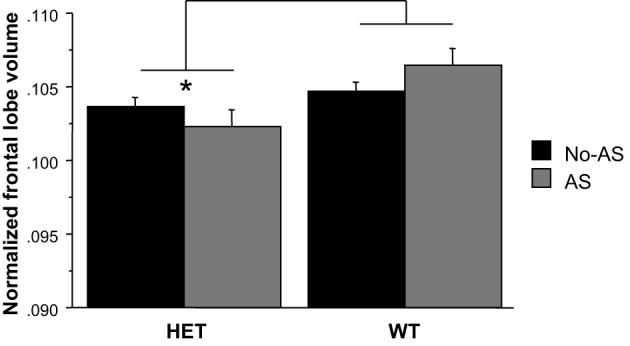
The region-of-interest analysis of the normalized frontal lobe volume demonstrated a smaller frontal lobe in HET relative to WT animals (^*^p < 0.008). Results are reported as mean ± s.e.m.

For the normalized frontal lobe volume [based on the population-specific atlas (Dorr et al., 2008)] there was a main effect of genotype [*F*_(1, 47)_ = 7.93, *p* < 0.008, Figure 9 and Table 2]: HET mice had a smaller frontal lobe volume compared to WT mice. Based on these findings, the frontal lobe was further examined on a voxel-wise basis in order to assess whether this structural difference was associated with a more localized structural change within the frontal lobe: no cluster survived multiple comparison correction (Supplementary Figure 2). RM-ANOVA of the left and right normalized HC volumes showed a significant AS × hemisphere interaction [*F*_(1, 47)_ = 6.8, *p* < 0.02, Table 2]: AS led to an increase in HC volume in the right hemisphere [*F*_(1, 47)_ = 4.4, *p* < 0.05] and not in the left hemisphere (*p* > 0.1), although this result did not survive Bonferroni correction for multiple comparison. The voxel-wise analysis is reported in Supplementary Figure 3; no cluster of volume expansion survived multiple comparison correction. There was no effect involving AS or genotype on amygdala (*p* > 0.2). The right AMY was greater in volume than the left AMY [*F*_(1, 47)_ = 181.6, *p* < 0.0001, Table 2].

## Discussion

In the present study, AS increased anxiety and decreased activity and did so to a similar degree in 5-HTT HET and WT mice. In addition, a genotype × stress interaction was found in the PRL task, where HET-AS mice achieved fewer reversals than did HET-No-AS mice. In terms of structural brain differences, AS reduced the volume of two long-range white matter tracts, the OT and the CP, in WT mice specifically. Moreover, AS was associated with increased HC volume and HET genotype with a decreased frontal lobe volume in a region-of-interest analysis.

### Short term effects of as and genotype on behavior

The stress procedure consisted of nine sessions of unpredictable, uncontrollable foot electro-shocks of progressively increasing intensity in a distinctive context remote from the home cage and cage mates. Electro-shock has been used extensively as an unconditioned stressor in rodent studies, including in demonstrations of the learned helplessness effect (Maier and Seligman, 1976; Pryce et al., 2011a, 2012). Repeated sessions were applied both to ensure stress exposure across adolescent development and based on previous evidence in humans and monkeys indicating that stressor reactivity increases as a function of repeated exposures and, furthermore, to a greater degree in *s* carriers (Caspi et al., 2003; Spinelli et al., 2012). For both experiments, No-AS mice exhibited decreased freezing across sessions suggesting habituation of neophobia. AS mice maintained the high level of freezing exhibited at session 1 across all subsequent sessions, consistent with contextual fear conditioning rather than habituation. In Experiment 1, AS mice also exhibited increased freezing at context re-exposure relative to No-AS mice, in the absence of genotype effects. In Experiment 2, the effect of AS on freezing to context at re-exposure was specific to HET-AS compared to HET-No-AS mice, with no AS effect in WT animals. Absence of a consistent genotype effect is in contrast to the increased freezing during fear conditioning observed in adult HET compared to WT mice (Pryce et al., 2012), a finding consistent with the increased reactivity to aversive stimuli observed in adult human *s* carriers (Pezawas et al., 2005). Since adolescence is a period of enhanced stress responsiveness in humans and animals (Lupien et al., 2009; Spear, 2009), it is possible that genotype differences associated with stress sensitivity are less consistently detectable in adolescence, and also that other environmental factors, such as social ranking in the home cage, increased within-genotype variance in stressor sensitivity.

### Long-term effects of as and genotype on behavior

Several research groups have investigated the effects of chronic or repeated stress during adolescence in rodents. Most of these studies have been conducted in rats and a variety of stress procedures have been used, both in terms of stressor type and the ages at onset and offset of the stressor. The general conclusion is that AS increases anxiety- and depression-related behaviors exhibited at testing in adulthood (Hollis et al., 2013; Mccormick and Green, 2013). The hyper-anxious phenotypes found consistently in adulthood after AS exposure (e.g., Schmidt et al., 2007) are relevant to the aetiopathogenesis of both human anxiety disorders and major depression, given that these conditions are highly co-morbid and that anxiety symptoms often start and develop earlier in life than depressive episodes (Kaufman and Charney, 2000).

In the present study, mice were tested for anxiety (NIH, EPM tests) and locomotion (OF test). In WT and HET mice, AS led to increased latency to eat a palatable food in the NIH test and reduced distanced traveled in the OF test, indicating development of increased anxiety and reduced locomotor activity, respectively. In HET mice specifically, AS led to decreased percentage of entries on the open arms indicating development of increased anxiety. Hyper-anxiety endophenotypes have been clearly reported in 5-HTT KO mice (i.e., main effects of genotype). However, in HET mice increased anxiety has not been found consistently, even in adulthood. For example, in a previous study investigating the effects of prenatal stress, HET mice showed increased time spent in the open arm suggestive of reduced anxiety (Van Den Hove et al., 2011), whereas another study showed a reduced number of open arm entries and head dips in HET compared to WT male mice in the EPM but no differences in the light-dark exploration test (Holmes et al., 2003). Given that AS led to some anxiogenic effects in HET mice specifically and full 5-HTT KO mice are anxious in the absence of stress, the current findings provide some support for the concept that full 5-HTT KO mice resemble HET-AS mice (Homberg and Van Den Hove, 2012).

Turning to the PRL task, HET-AS mice completed less reversals and needed more time to complete the session (i.e., obtain all available rewards) compared to HET-No-AS mice, with WT mice exhibiting intermediate performance between the two HET groups. The increased session duration of HET-AS mice was associated with an increased number of trials with long post-reinforcement latency to make an operant response after delivery of the previous reward. This relatively high number of trials with long post-reinforcement latency likely contributed to the lower number of reversals completed: such long intervals put greater demand on working memory and have a higher probability of leading to a reward-shift error on the next trial; if such an interval occurs and leads to an error when the mouse is close to the reversal criterion (8 consecutive correct trials) then this will have a higher impact on number of reversals completed than the more common reward-shift errors made just after reversal. There are two possible explanations for these long post-reinforcement latency trials. Firstly, HET-AS mice could have been less motivated to perform the task. However, on two other motivation measures, namely the number of feeder responses and the latency to collect the reward, no differences were found. Second, HET-AS mice could have been more sensitive to the high response-reinforcement uncertainty associated with the PRL task. Although the long post-reinforcement latency did not occur disproportionately often after false negative feedback or an incorrect response, reward expectancy is the major determinant for the number of reversals completed (Ineichen et al., 2012) and it is therefore possible that increased sensitivity to uncertainty in HET-AS mice was expressed as a long post-reinforcement latency.

The PRL tasks findings of reduced reversals and increased post-reinforcement pause in HET-AS relative to HET-No-AS mice are particularly interesting in light of the reports that PRL performance was reduced in *s*/*s* adolescents exposed to childhood stressful life events and also in patients with major depression (Murphy et al., 2003; Taylor Tavares et al., 2008; Owens et al., 2012). Caution is required when comparing human and mouse versions of PRL tasks, of course. In these human studies a visual discrimination task is used while the rodent version uses a spatial discrimination task. More importantly, human subjects likely deploy a trial-by-trial decision making strategy whereas mice assess on-going average reward expectancy [discussed in detail in Ineichen et al. (2012)]. This trial-by-trial strategy is also important when considering the performance impairments reported in subjects with major depression and *s*/*s* adolescents exposed to childhood stressful events, as indicated by the fact that both groups show an increased likelihood to shift specifically after a misleading negative feedback, which was not the case in HET-AS mice. Despite these differences, the current findings suggest that HET mice exposed to AS may be more sensitive to performance uncertainty. Although the behavioral response to ambiguous feedback is likely to differ in humans and rodents (increased negative feedback sensitivity vs. increased post-reinforcement latency), intolerance of uncertainty is found in major depression and anxiety disorders (Carleton et al., 2012; Boswell et al., 2013) and recent evidence indicates that the 5-HTTLPR also influences decision making under ambiguous circumstances (He et al., 2010; Stoltenberg and Vandever, 2010; Stoltenberg et al., 2011). Moreover, *s/s* adolescents exposed to an adverse childhood environment also show decreased performance during neutral (considered more ambiguous) trials in an emotional Go/No-Go task compared to *s/s* carriers not exposed to childhood adversity (Owens et al., 2012). Importantly, a negative bias in the interpretation of ambiguous stimuli has recently been shown in rats exposed to chronic unpredictable stress during adolescence (Chaby et al., 2013). These findings suggest that increased sensitivity to uncertainty may mediate the PRL deficits reported in humans and mice. It is also noteworthy that HET-AS mice exhibited increased anxiety in the EPM relative to HET-No-AS mice, and that both the EPM test and PRL task require resolution of a two-choice conflict under conditions of emotional demand. There was no effect of HET genotype on acquisition of serial reversal learning; this replicates a previous study under identical conditions (Ineichen et al., 2012). However, it is noteworthy that there are reports that HET mice exhibit enhanced reversal learning relative to WT (Brigman et al., 2010), and that 5-HTT KO in rat (Nonkes et al., 2011, 2013) and low 5-HTT gene function in rhesus macaque (Vallender et al., 2009; Jedema et al., 2010) are both associated with enhanced reversal learning.

#### Long term effects of as and genotype on brain structures

Stress during development has been shown to induce a cascade of physiological, neurochemical, and hormonal changes that can lead to long-term structural and functional alterations of the PFC, AMY, and HC and their white matter connections (Teicher et al., 2003; Mccrory et al., 2011). Investigating the effects of AS and 5-HTT genotype on these regions specifically using a region-of-interest analysis, AS was associated with increased HC volume and HET with reduced frontal lobe volume, and there was no effect of either factor in AMY. In human studies and animal experimental studies, exposure to stressful life events during early development is often associated with reduced development of HC volume (Teicher et al., 2003; Andersen and Teicher, 2004; Mccrory et al., 2011; Hart and Rubia, 2012). Importantly, although other factors are likely to play a role, decreased HC volume is usually associated with increased corticosteroid levels (Isgor et al., 2004). This is consistent with the neurotoxicity hypothesis, which postulates that prolonged stress-induced hyper-corticoidism leads to reduced neurogenesis and apoptosis. However, we found no effect of AS or genotype on plasma CORT titers. It is possible that AS led to a transient increase in plasma CORT, since increased baseline CORT levels have been reported in adult mice 1 week after 7-week social stress conducted across adolescence and early adulthood (Schmidt et al., 2007), but when a large cohort of mice was tested 5 weeks after the 7-week stress, only a subgroup of animals still showed increased CORT (Schmidt et al., 2010). Importantly, AS mice exhibited hyper-anxiety in the NIH test and reduced locomotion in the OF test. Given that both of these tests are HC-dependent (Winograd and Viola, 2004; Bechtholt et al., 2008), the HC volume increase exhibited by AS mice irrespective of 5-HTT genotype could have contributed to these behavioral effects. Therefore, increased HC volume may indicate an abnormal HC development and possibly an increased vulnerability to further stress exposure. This explanation is supported by recent findings in rodents. Mccormick et al. (2012) showed that adult male rats exposed to social instability in adolescence demonstrated increased expression of proteins associated with synaptic plasticity but reduced performance in HC-dependent memory tasks (Mccormick et al., 2012). Moreover, Barha et al. (2011) found that a 3-week chronic restrain stress in adolescence did not affect basal CORT levels but induced a slight increase in HC cell survival in male rats. In contrast, in females stress increased basal CORT and reduced neurogenesis, indicating that the long-term consequences of adolescence stress can be sex-specific (Barha et al., 2011).

In humans and other primates, the *s* allele has been associated with decreased volume of the AMY and the prefrontal, temporal and parietal cortices (Canli et al., 2005; Pezawas et al., 2005; Jedema et al., 2010; Selvaraj et al., 2011). Consistent with these findings, HET mice showed reduced frontal lobe volume (using a region-of-interest analysis) and a volume contraction of the right temporal-parietal cortex (using a whole brain voxel-wise analysis) when compared to WT mice. Since the most consistent finding across primate studies is decreased volume of the anterior cingulate cortex in *s* carriers, the frontal lobe was further examined on a voxel-wise basis to assess for more localized structural changes in the medial part of the prefrontal lobe; several small clusters were found, none of which survived multiple comparison correction (see Appendix). In contrast to our hypothesis, we did not detect any AS affect on the frontal lobe.

In addition, the whole brain analysis showed a genotype × AS interaction in two white matter tracts, the CP and OT. In WT mice specifically, AS decreased CP and OT volumes, whereas in HET mice AS had no significant effect. Data in humans and animals consistently demonstrate effects of developmental stress on white matter structures, including the corpus callosum (Teicher et al., 2003; Daniels et al., 2013). In humans, adolescence is considered an important period for the development of long range connections (Hagmann et al., 2010), which is also associated with an increase in functional connectivity and improvement in cognitive abilities (Paus, 2005, 2010; Fair et al., 2007). Although developmental changes of the mouse brain have not been widely investigated using MRI, preliminary data indicate that total brain volume reaches adult size by the third week of life and that maturational changes, including measures associated with white matter integrity, continue throughout adolescence (Larvaron et al., 2007; Chuang et al., 2011). Since the CP contains all the cortical projections to the pons, including corticofugal fibers that project from the cerebral cortex to the cerebellum (Allegrini and Wiessner, 2003; Ramnani et al., 2006), our results suggest that juvenile stress affected long-distance white matter connections in WT mice specifically. Measures of functional and structural connectivity, such as resting state functional MRI or diffusion tensor imaging, will be required to provide insights into which connections were more affected. These white matter changes are consistent previous studies in rats reporting reduced fractional anisotropy in the genu of the corpus callosum in 5-HTT KO relative to WT rats (Van Der Marel et al., 2013), and altered callosal axon myelination by transient inhibition of 5-HTT function with selective serotonin reuptake inhibitors during the perinatal period (Simpson et al., 2011). Moreover, reduced fractional anisotropy in various white matter structures including the CP has been reported in depressed patients carrying vulnerability polymorphisms of other genes (Murphy et al., 2012). Reduced fractional anisotropy in the CP has also been reported in young adolescents exposed to neglect during childhood (Hanson et al., 2013) and in schizophrenia (Ashtari et al., 2005; Cheung et al., 2008; Davenport et al., 2010; Koch et al., 2010), a disorder also associated with abnormal brain maturation during adolescence and exposure to stressful life events during development (Holtzman et al., 2013).

Although the results of the present study indicate that AS had some long-term effects on behavior and brain structure, these effects were moderate and specific. It is currently unclear whether adolescent mice are generally resilient to stressors or whether the effects were limited due to stress being experienced in relatively short time episodes and in a distinctive context remote from the home cage which was the major environment and was non-stressful. Adolescent psychosocial stress in the home cage e.g., an adaptation of chronic social defeat could exert more effects on brain-behavior development. In addition, we cannot exclude that some of our findings (e.g., increased HC volume) conferred increased resilience to further stress exposure. Repeated exposure to predator stress in adolescence, for example, has been shown to increase resilience rather than anxiety- and depression-related behaviors if rats were housed with conspecifics during the stress exposure (Kendig et al., 2011). The type of stress procedure may also be important when considering the effects of (or lack thereof) genotype. Previous findings in adult rats show that whereas 5-HTT KO led to increased anxiety- and depression-related behaviors, absence of 5-HTT was protective against construction stress (Schipper et al., 2011). In humans, the risk for major depression in *s* allele carriers is particularly associated with both early life stress and repeated stress exposures, suggesting that stress will be experienced across childhood and adolescence in these subjects (Karg et al., 2011), thus it is possible that negative interaction effects associated with reduced 5-HTT expression are greater for stressors occurring across the pre- or post-weaning period or indeed prenatally. An additional limitation of the present study is that we only investigated male mice but previous studies show important sex differences in response to stress exposure and 5-HTT genotype (e.g., Holmes et al., 2003; Barha et al., 2011; Van Den Hove et al., 2011). Moreover, mice were exposed to repeated testing and the findings may have been influenced by the prior experience. Finally, we did not find significant correlations between behavioral measures and regional brain volumes (data not shown). However, regional brain volumes measured in adulthood could only be correlated with behavioral measures acquired during early and late adolescence (stress procedure, context re-exposure, and OF) because all other behavioral results were collected in a separate group of animals.

In conclusion, repeated stress exposure during adolescence had long term effects on behavior and structural brain development. AS led to increased anxiety and decreased locomotor activity, in the absence of effects on HPA reactivity. In addition, in HET mice specifically AS impaired behavior in the PRL task: HET-AS mice showed a decreased number of reversal completed and an increased post reinforcement latency compared to HET-No-AS mice, suggesting that HET mice exposed to AS develop increased sensitivity to environmental uncertainty. In terms of structural brain differences, AS was associated with decreased volume of the long-range white matter tracts OT and CP in WT mice specifically. In addition, the region-of-interest MRI analysis showed that AS was associated with an increased HC volume and the HET genotype with a decreased frontal lobe volume. Future studies investigating the functional significance of these structural findings are warranted.

### Conflict of interest statement

The authors declare that the research was conducted in the absence of any commercial or financial relationships that could be construed as a potential conflict of interest.
