# Supplementary material for: Effects of repeated adolescent stress and serotonin transporter gene partial knockout in mice on behaviors and brain structures relevant to major depression
Source: Front Behav Neurosci. 2013 Dec 31;7:215. doi: 10.3389/fnbeh.2013.00215 (PMC3876674; doi:10.3389/fnbeh.2013.00215)

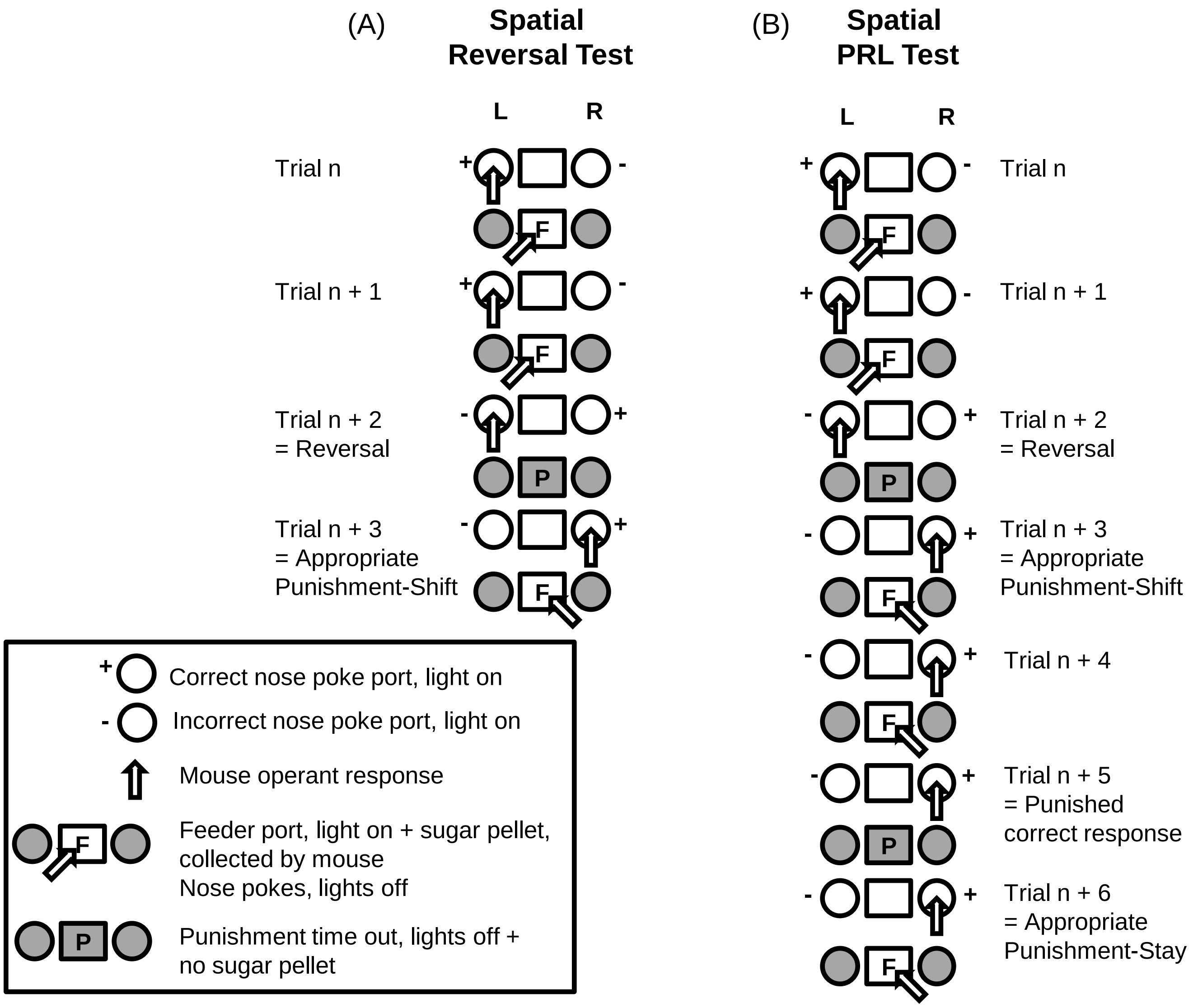

Supplemental Figure 1. Schematic for the layout and procedure for (A) the spatial reversal test and (B) the spatial probabilistic reversal learning test. L and R indicate left and right nose-poke ports, respectively.

Supplemental figure 2

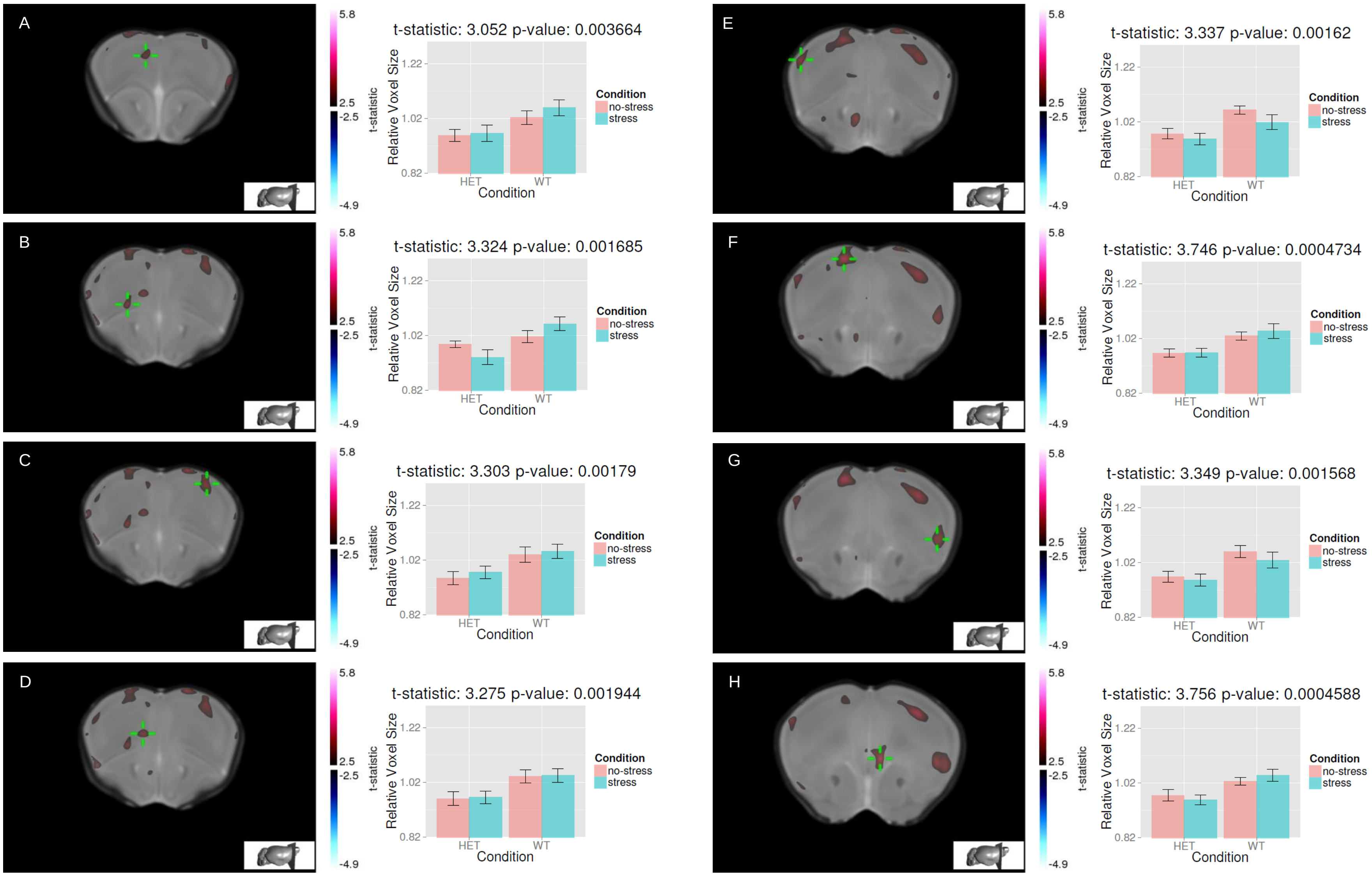

Supplemental figure 2

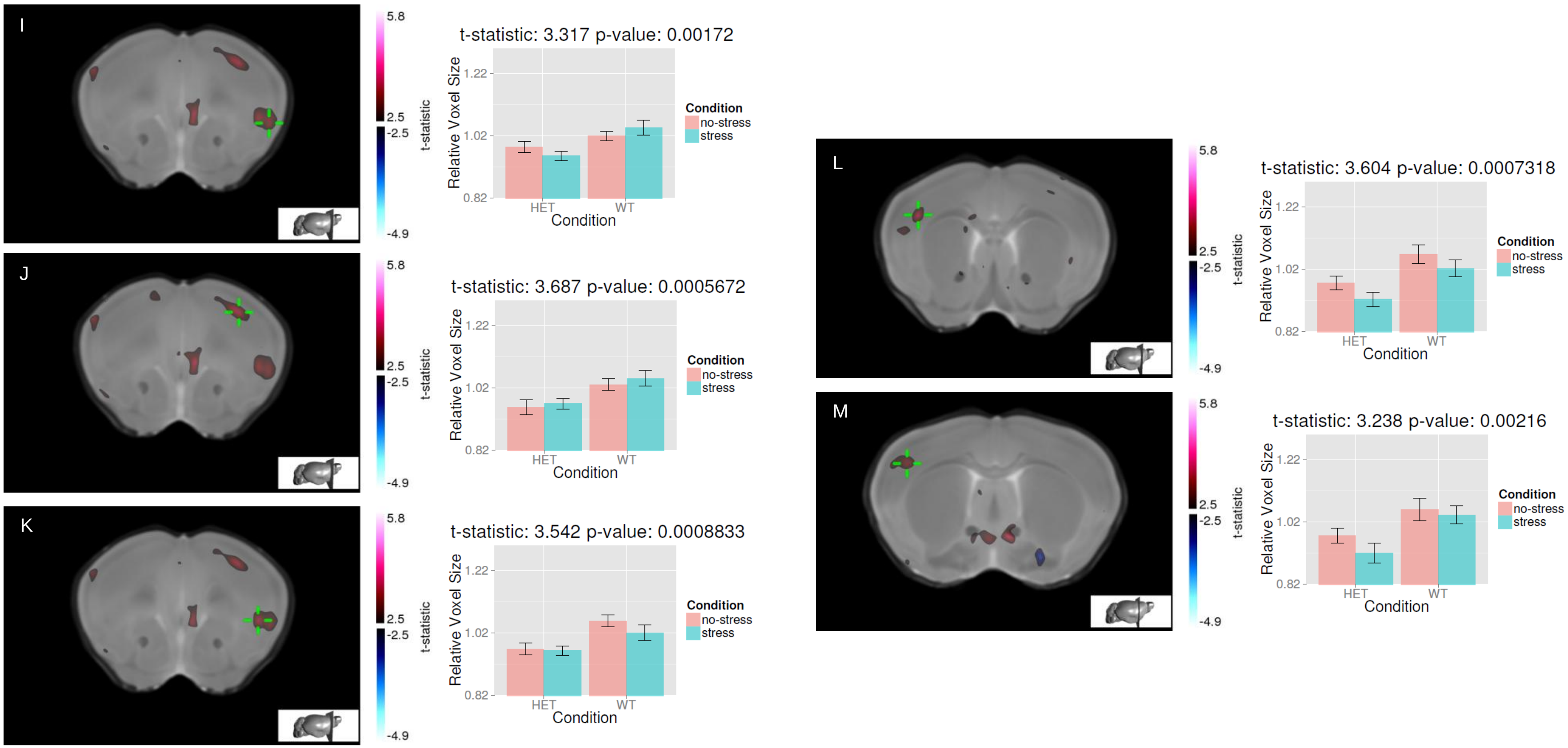

Supplemental figure 3

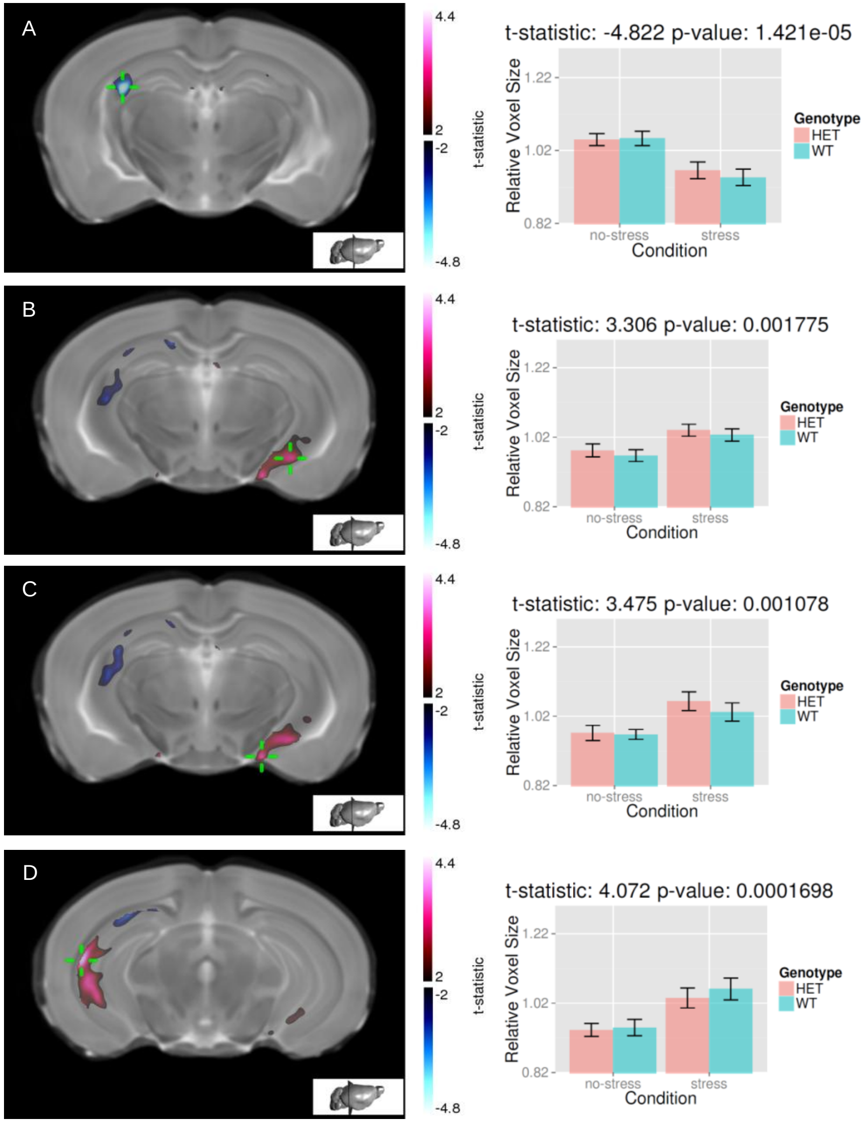

Supplement: Supplementary file 1 [file Presentation1.PDF]
